# Remote Text‐Supplemented Audiobook Intervention Supports Children's Explicit and Incidental Vocabulary Learning

**DOI:** 10.1111/desc.70159

**Published:** 2026-03-17

**Authors:** Halie A. Olson, Ola Ozernov‐Palchik, Xochitl M. Arechiga, John D. E. Gabrieli

**Affiliations:** ^1^ McGovern Institute for Brain Research Massachusetts Institute of Technology Cambridge Massachusetts USA; ^2^ Brain and Cognitive Sciences Massachusetts Institute of Technology Cambridge Massachusetts USA; ^3^ Wheelock College of Education & Human Development Boston University Boston Massachusetts USA

**Keywords:** audiobooks, independent reading, randomized controlled trial (RCT), remote, socioeconomic status (SES), vocabulary

## Abstract

Vocabulary knowledge is foundational to educational success, but significant gaps exist between students with reading disabilities or those from disadvantaged backgrounds and their peers. These gaps have cascading effects, as children with lower vocabulary knowledge are less likely to acquire new words through independent reading and are less responsive to vocabulary instruction methods like read‐alouds and explicit teaching. The effectiveness of explicit instruction relies on individualization, which typically places substantial demands on educators and thereby hinders the adoption of evidence‐based methods. A potential solution is using audiobooks supplemented by explicit and individualized remote instruction from paraprofessionals. We conducted a randomized controlled trial (RCT) intervention study in which children listened to text‐supplemented audiobooks, either alone or with scaffolded instructional support. Third and fourth‐grade students (*N* = 314, age: mean (SD) = 9.47(0.57) years) were randomly assigned to one of three conditions (Audiobooks‐Only, Audiobooks+Scaffold, or active control) for 8 weeks. Participants in the two audiobook intervention groups showed significant improvements in book‐specific vocabulary, while the active control group showed no improvement. The effectiveness of the intervention varied by reading ability and socioeconomic status (SES): poor readers benefited only when audiobooks were paired with one‐on‐one scaffolding, whereas children from lower‐SES backgrounds showed modest, nonsignificant gains from audiobook access alone and did not experience additional benefits from scaffolding. Additionally, the Audiobooks+Scaffold group spent more time listening to recommended audiobooks during the study. These findings suggest that text‐supplemented audiobooks, particularly when combined with personalized support, can be a valuable tool for supporting vocabulary development in struggling readers.

**Summary:**

- Children successfully learned new vocabulary words by engaging with text‐supplemented audiobooks.
- Vocabulary gains were largest amongst students who additionally received one‐on‐one remote scaffolding sessions throughout the intervention period.
- Poor readers only benefited when text‐supplemented audiobooks were paired with one‐on‐one instructional support.
- Students from lower socioeconomic backgrounds showed smaller, nonsignificant gains from either component, suggesting a need for additional support to achieve comparable vocabulary growth.

## 1 Introduction

### 1.1 Vocabulary Knowledge and Reading Proficiency

Vocabulary knowledge is widely recognized as the single best predictor of reading achievement and school completion (Hoff 2013; Ricketts et al. 2007; Sénéchal et al. 2006). Vocabulary acquisition occurs both incidentally—through multiple exposures to words across various contexts, as individuals use probabilistic cues to infer the meaning of words based on the context in which those words are encountered (Stahl and Nagy 2007)—and via direct instruction, when caregivers, teachers, and others explicitly instruct vocabulary (Loftus and Coyne 2013). It has been estimated that the majority of words are acquired incidentally early in development through exposure to the oral linguistic environment, and during school years and beyond through independent reading (Nagy et al. 1985).

Reading books provides an opportunity for children to encounter more complex vocabulary and language than they are exposed to in everyday speech (Montag 2019; Montag et al. 2015). Repeated encounters with new words in meaningful text contexts are a key driver of vocabulary growth, leading to a richer lexicon. A robust lexical base, in turn, supports text comprehension by creating a semantic framework from which readers can more readily infer unfamiliar word meanings (Perfetti 2007). Furthermore, frequent reading enhances decoding skills, enabling children to tackle more advanced materials and fostering the development of more comprehensive content and linguistic knowledge (Spear‐Swerling et al. 2010; Stanovich 2000). Such cumulative knowledge allows learners to flexibly integrate word meanings into novel contexts and across multiple representations (Reichle and Perfetti 2003). Therefore, reading frequency is crucial for sustaining vocabulary expansion in primary school.

However, children vary widely in the amount of time they spend reading. In particular, struggling readers are often less motivated to spend their free time reading (Anderson et al. 1988; Mol and Bus 2011; van Bergen et al. 2018, 2023). Differences in independent reading can lead to reciprocal and cumulative gaps in vocabulary growth based on mere differences in exposure to new and advanced vocabulary (Acheson et al. 2008; Biber 1991; Duff et al. 2015; Montag et al. 2015; Montag and MacDonald 2015). More limited exposure to words leads to shallower lexical knowledge, which in turn impedes comprehension of text and limits the ability to infer word meanings from semantic context. Additionally, because decoding and comprehension require greater cognitive effort in struggling readers, fewer resources are available for integrating new words, further hindering incidental vocabulary learning. Together, differences in reading experiences lead to cascading gaps in vocabulary knowledge that continue throughout development and into adulthood. Formal schooling experiences have a limited impact on closing these gaps, often leaving children who start school with low vocabulary levels at a continued disadvantage (Catts et al. 2006; Christian et al. 2000; Cunningham and Stanovich 1997; Stanovich 1986).

### 1.2 Voluntary Reading Programs

Based on the link between reading habits and vocabulary knowledge, a simple solution would seem to be encouraging independent reading; however, efforts to promote independent reading among children, both during and outside of school hours, have generally shown minimal or no impact on reading proficiency (Erbeli and Rice 2022; National Reading Panel [US] & National Institute of Child Health, & Human Development [US], 2000). A study examining children in Grades 3–5 assessed the impact of a voluntary summer reading program, both alone and combined with explicit instructional support (Kim and White 2008). The study found that providing books aligned with students' reading abilities and interests, coupled with reading reminders for children and caregivers, notably increased reading frequency, particularly among minority students. However, enhancements in reading skills were only observed in the group that received additional scaffolded support, which included comprehension strategies and oral reading practice from teachers and parents.

### 1.3 Explicit Instruction

A critical feature of effective educational programs for improving student outcomes, particularly for students with disabilities, is explicit instruction (Denton et al. 2003; Gersten et al. 2001, 2020; Swanson and Hoskyn 1998; Vaughn et al. 2000). Educational guidelines have increasingly emphasized the importance of incorporating explicit vocabulary instruction into school curricula (National Governors Association Center for Best Practices and Council of Chief State School Officers 2010; National Reading Panel [US] & National Institute of Child Health, & Human Development [US], 2000). A synthesis of studies on vocabulary interventions suggests that the most effective instruction provides multiple encounters with words across varied contexts, addresses multiple word meanings, and involves active processing beyond simple definitions (Wright and Cervetti 2017). Furthermore, different instructional components uniquely influence the breadth and depth of children's word knowledge (Ard and Beverly 2004; Coyne et al. 2009; Penno et al. 2002; Sénéchal 1997). For example, repeated readings of a text help learners encode, associate, and store novel information, thereby enhancing receptive vocabulary—the ability to accurately recognize and understand target words (Sénéchal 1997). In contrast, active processing strategies, such as providing explicit definitions or breaking down the morphology of new words, further support expressive vocabulary by enabling children to define words accurately and use them appropriately in various contexts. Researchers have long argued that to expand children's language, “decontextualized” discourse—beyond everyday conversational topics—is crucial (Heath 1983; Snow and Dickinson 1991). This type of contextual expansion is notably accomplished through methods like “read‐alouds,” which stretch children's comprehension by exposing them to richer language and more complex subject matter (Beck and McKeown 2001; Snow 2002).

For struggling readers, such approaches are most effective in small groups or one‐on‐one with instruction tailored to their learning needs (Coyne et al. 2007; Fien et al. 2011). However, these methods are resource‐heavy and hard to implement widely in schools (e.g., Baker et al. 2015; Kim et al. 2021). Therefore, despite the proven effectiveness of these approaches, vocabulary instruction remains infrequent and unsystematic in many school curricula (Biemiller 2001; Durkin 1978; Lesaux et al. 2010; Scott and Nagy 1997). Furthermore, there is little evidence regarding whether these effects can be sustained long‐term without a child's independent reading engagement.

Taken together, these previous studies have identified multiple exposures to vocabulary words through independent reading and explicit vocabulary instruction as a significant contributor to children's vocabulary growth. In the current study, we evaluated whether text‐supplemented audiobooks, supported by one‐on‐one scaffolded instruction, could improve children's vocabulary knowledge via a remote intervention program. Text‐supplemented audiobooks offer access to advanced reading materials without the initial barrier of print decoding skills, thereby increasing exposure to complex language and vocabulary (Wolfson 2008). Some evidence suggests that audiobooks, along with text‐to‐speech or read‐aloud tools, may support comprehension and vocabulary learning, particularly in younger children and children with reading difficulties (Singh and Alexander 2022; Wood et al. 2018), though this approach has been underexplored.

### 1.4 Remote Intervention Context

The Covid‐19 pandemic accelerated the adoption of remote research methodologies, highlighting their potential to reach a more diverse population and implement studies in a more naturalistic and reproducible manner (Bambha and Casasola 2021; Liu et al. 2021; Morini and Blair 2021; Ozernov‐Palchik et al. 2022; Tsuji et al. 2022). Despite the growing interest in remote interventions, the effectiveness of remote vocabulary interventions has not been systematically investigated. Vocabulary interventions have traditionally been implemented in person, often within classroom settings or through parent‐child interactions at home (Cervetti et al. 2023). With the shift to remote learning, there was an opportunity to explore how such interventions could be adapted to virtual formats. Potential benefits of remote intervention approaches include increased accessibility for children who may not have consistent access to reading support (e.g., due to geographic or socioeconomic barriers), as well as increased access to personalized structured support. Moreover, given the scarcity of teacher time, supplementing in‐person whole classroom instruction with individualized online instruction from paraprofessionals or other individuals with less specialized training presents a significant opportunity for providing additional explicit support to students in need.

Notably, the Covid‐19 pandemic also exacerbated socioeconomic disparities in education, disproportionately impacting students from lower socioeconomic (SES) backgrounds (Alejo et al. 2024; Engzell et al. 2021; Gee et al. 2023; George et al. 2021; Peters et al. 2025). Even before these impacts were known, prior research on education disruptions indicated that lower‐SES students may be at higher risk for learning loss. Over summer vacations, lower‐SES students often lose ground on reading compared to their higher‐income peers (e.g., Cooper et al. 1996; cf., von Hippel et al. 2018), as do children with reading difficulties (Christodoulou et al. 2017). Indeed, summer reading interventions may be particularly beneficial for low‐income children (Kim and Quinn 2013; Romeo et al. 2018). Therefore, we made a concerted effort to recruit a diverse sample with respect to socioeconomic status (Ozernov‐Palchik et al. 2022). We hypothesized that students from lower‐SES backgrounds may especially benefit from a reading‐based intervention during the disruptive time of the pandemic.

### 1.5 Current Study and Hypotheses

In the current study, we targeted vocabulary knowledge in 3rd and 4th‐grade students through a randomized controlled trial (RCT) intervention. The first aim of the RCT was to determine whether voluntary listening to text‐supplemented audiobooks provided by *Learning Ally* would facilitate implicit vocabulary learning. Audiobooks remove text decoding as a barrier to reading, exposing children to narrative structure, new vocabulary, and syntactic complexity that are crucial to reading comprehension, but are not always found in everyday speech (Massaro 2015). Thus, we hypothesized that reading text‐supplemented audiobooks that introduced new words and appropriately challenging content would improve children's vocabulary scores. The second aim was to determine whether providing children with explicit instructional support (i.e., scaffolding) would enhance the efficacy of a text‐supplemented audiobook intervention. In addition to listening to audiobooks, we randomly assigned one group of children to receive one‐on‐one instructional support from college students trained to implement effective strategies for explicit vocabulary instruction (Beck and McKeown 2007; Coyne et al. 2007, 2009) as well as a validated curriculum for comprehension strategies (Language and Reading Research Consortium et al. 2014; Language and Reading Research Consortium (LARRC), 2016; Language and Reading Research Consortium (LARRC), Johanson, et al. 2016; Language and Reading Research Consortium (LARRC) et al. 2019). We hypothesized that this explicit instruction would significantly enhance the benefits of the text‐supplemented audiobook intervention, especially for children who are poor readers. In the present study, “poor readers” refers to children scoring at or below the 20th percentile on a standardized measure of passage reading fluency. Although comprehension depends on multiple cognitive and linguistic abilities (Catts 2022; Kendeou et al. 2016; Scarborough 2001; Seigneuric et al. 2000), we focused on decoding fluency because we predicted that audiobooks with scaffolding would be most effective for children whose decoding difficulties limit access to rich vocabulary through independent reading.

To summarize, the theory of change guiding this research proposes that engagement with text‐supplemented audiobooks, by removing the barriers of decoding, supports children's incidental learning of new word meanings and leads to improvements in vocabulary knowledge. Direct instruction in word meanings further supports children's vocabulary acquisition through explicit learning. Since previous literature has primarily shown the short‐term efficacy of interventions on proximal outcomes rather than standardized measures of vocabulary and comprehension (Wright and Cervetti 2017), we included both proximal measures (assessing learning of vocabulary specifically encountered in the text‐supplemented audiobooks) and standard measures of vocabulary. We expected to see larger gains on the proximal outcome measures than on the distal ones.

## 2 Methods

### 2.1 Design

**FIGURE 1:**
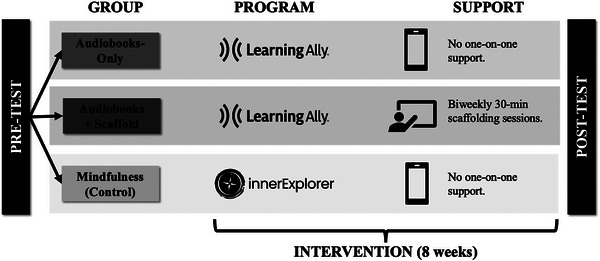
Schematic of study design.

We employed an RCT study design (Figure 1). Participants were assigned to one of three intervention conditions based on a block randomization procedure (i.e., groups assigned based on a pre‐generated block‐randomized sequence (integers 1‐3). The intervention conditions included: Audiobooks‐Only, Audiobooks+Scaffold, and Mindfulness (the active control group). Each intervention lasted 8 weeks and was conducted entirely remotely. Testing was also conducted remotely before (pre‐test) and after (post‐test) the intervention. All outcome measures were collected at both pre‐test and post‐test; additional measures were typically collected at pre‐test. We used an intent‐to‐treat approach and accounted for missing data in the data analysis. We note that the study was conducted during the Covid‐19 pandemic, with data collection beginning in mid‐summer 2020 and concluding in spring 2022.

### 2.2 Participants

314 children participated in this study (see Table 1 for participant characteristics). Participants were primarily recruited via school partnerships and online advertising (see Ozernov‐Palchik et al. 2022 for additional details). To be eligible for the study, children had to be in or entering third or fourth grade at the time of recruitment, fluent in English, have a parent or guardian that spoke English or Spanish, have normal or corrected to normal hearing and no diagnosis of autism spectrum disorder according to parental report, and have a nonverbal reasoning standard score of 80 or above on the Kaufman Brief Intelligence Test administered during pre‐test (KBIT‐2; Kaufman and Kaufman 2004) to rule out a broad cognitive difficulty. Parents or legal guardians provided informed consent, and children provided verbal assent. Children were compensated $20 per hour for all pre‐testing and post‐testing sessions, and caregivers were additionally compensated $5 per survey for completing a total of 10 surveys at the beginning and end of the intervention period. Study procedures were approved by MIT's Committee on the Use of Humans as Experimental Subjects. Detailed experimental procedures and considerations for online RCTs with developmental populations were previously published in Ozernov‐Palchik et al. 2022.

**TABLE 1:** Participant characteristics.

	Audiobooks‐only	Audiobooks+Scaffold	Mindfulness	Overall	Group differences
*n*	105	108	101	314	
Age (years)	*M*(*SD*) = 9.44 (0.51), range: 8.32–10.48	*M*(*SD*) = 9.43 (0.62), range: 8.05–10.79	*M*(*SD*) = 9.54 (0.55), range: 8.01–10.79	*M*(*SD*) = 9.47 (0.57), range: 8.01–10.79	*F*(2,311) = 1.19, *p* = 0.304
Gender	female = 44; male = 61	female = 52; male = 56	female = 48; male = 53	female = 144; male = 170	*χ* ^2^(2) = 1.00, *p* = 0.61
Race/ethnicity	Asian = 9; Black/African American = 5; Hispanic/Latino = 15; White = 43; Multiple = 21; *NA* = 12	Asian = 9; Black/African American = 0; Hispanic/Latino = 21; White = 45; Multiple = 24; *NA* = 9	Asian = 7; Black/African American = 3; Hispanic/Latino = 12; White = 51; Multiple = 21; *NA* = 7	Asian = 25; Black/African American = 8; Hispanic/Latino = 48; White = 139; Multiple = 66; *NA* = 28	*χ* ^2^(10) = 9.77, *p* = 0.46
KBIT matrices	*M*(*SD*) = 109.07 (14.93), range: 70–145	*M*(*SD*) = 107.08 (14.50), range: 77–135	*M*(*SD*) = 108.25 (11.12), range: 84–137	*M*(*SD*) = 108.12 (13.64), range: 70–145	*F*(2,311) = 0.57, *p* = 0.57
DIBELS PRF	*M*(*SD*) = 47.47 (33.54), range: 0–99	*M*(*SD*) = 48.40 (30.69), range: 0–98	*M*(*SD*) = 47.84 (33.76), range: 0–99	*M*(*SD*) = 47.91 (32.56), range: 0–99	*F*(2,311) = 0.02, *p* = 0.98
Poor Reader	Yes = 31 (No = 74) Higher‐SES: Yes = 18, No = 51 Lower‐SES: Yes = 13, No = 23	Yes = 30 (No = 78) Higher‐SES: Yes = 14, No = 56 Lower‐SES: Yes = 16, No = 22	Yes = 30 (No = 71) Higher‐SES: Yes = 13, No = 50 Lower‐SES: Yes = 17, No = 21	Yes = 91 (No = 223) Higher‐SES: Yes = 45, No = 157 Lower‐SES: Yes = 46, No = 66	*χ* ^2^(2) = 0.12, *p* = 0.94
Parent Avg Education (years)	*M*(*SD*) = 15.89 (2.82), range: 6–21	*M*(*SD*) = 15.88 (2.98), range: 6–21	*M*(*SD*) = 16.09 (2.78), range: 8–21	*M*(*SD*) = 15.95 (2.86), range: 6–21	*F*(2,311) = 0.19, *p* = 0.83
Grade	Grade 3 = 34 Grade 4 = 70 Grade 5 = 1	Grade 3 = 36 Grade 4 = 71 Grade 5 = 1	Grade 3 = 27 Grade 4 = 74	Grade 3 = 97 Grade 4 = 215 Grade 5 = 2	*χ* ^2^(4) = 2.31, *p* = 0.68

*Note*: Table includes imputed data, with the exception of race/ethnicity, KBIT matrices, and grade. KBIT matrices are standard scores (normed by age, centered at 100 with a standard deviation of 15; Kaufman and Kaufman 2004). DIBELS PRF are percentile scores (normed by grade; Good and Kaminski 2002). Poor Reader is a measure of reading decoding based on DIBELS PRF (≤20th percentile; Good and Kaminski 2002).

### 2.3 Attrition

Of the 314 participants assigned to intervention groups, 54 did not complete post‐testing, resulting in an overall attrition rate of 17.2%. Attrition rates were 17.1% in the Audiobooks‐Only group, 14.8% in the Audiobooks+Scaffold group, and 19.8% in the Mindfulness group.

### 2.4 Intervention

Each participant was randomly assigned to an intervention group for an approximately 8‐week period (in extenuating circumstances, the intervention period was occasionally longer than 8 weeks to fit families’ schedules). The start dates were rolling, such that a participant's start date was always on a Monday. The intervention conditions are described below. For more details on the coordination and administration of the intervention conditions remotely, please see Ozernov‐Palchik et al. 2022.

Audiobooks‐Only: Participants in the Audiobooks‐Only condition were given access to the *Learning Ally* platform, which contains a library of audiobooks along with text that can be accessed via computer, smartphone, or tablet. As the book is read aloud, the words are highlighted on the screen. Each participant's account had a set of recommended books selected based on their listening comprehension level, operationalized based on their CELF Understanding Spoken Paragraph scores (see Table S1 for lists of books and corresponding CELF scores; see overview of CELF and other assessments below). The goal was to curate a selection of books in each child's zone of proximal development (Vygotsky and Cole 1978; cf., Kim and Guryan 2010)—that is, slightly challenging based on the child's current listening comprehension level, but that the child could access with support. There were three “tracks” of curated books (drawing from 26 titles selected for the study), and all tracks included fiction and nonfiction options, as well as diverse characters. Participants were asked to listen to their books for approximately 90 min per week. They were instructed to listen to one book at a time and were typically given a choice between two books each time they finished a book, with the exception of the first and last book, which we aimed to standardize between all children at a particular level.

Audiobooks+Scaffold: This condition was identical to the Audiobooks‐Only condition, with the addition of twice‐per‐week one‐on‐one “scaffolding sessions” focused on vocabulary and reading comprehension. Each participant in the Audiobooks+Scaffold condition was assigned to an undergraduate student who served as their “learning facilitator” for the study. Participants in the Audiobooks+Scaffold group met with their learning facilitator one‐on‐one via Zoom for two 30‐min sessions per week for the duration of the intervention. During these sessions, learning facilitators were asked to do three things. First, they checked in with the student about reading progress, identified barriers to reading, and brainstormed suggestions as needed (duration: ∼2–5 min). Next, learning facilitators taught two vocabulary words from the text, from a pre‐selected list of vocabulary words identified for this study (see Proximal Vocabulary Assessments below for how we selected target words). The vocabulary component of the session was based on strategies from Beck and McKeown 2007, such as asking the child to connect the word to their own experiences and giving examples in contexts other than the one used in the book (see OSF for more example strategies; duration: ∼5–10 min). Learning facilitators also administered Proximal Vocabulary Assessments when a child started or finished a book, except for the first and final book assessments, which were administered by experimenters blind to group assignment (see **Outcome Measures** below). Finally, learning facilitators taught and reviewed a reading comprehension strategy using a lesson plan based on Language and Reading Research Consortium curriculum (Language and Reading Research Consortium et al. 2014; Language and Reading Research Consortium (LARRC), 2016; Language and Reading Research Consortium (LARRC), Johanson, et al. 2016; Language and Reading Research Consortium (LARRC) et al. 2019), see Table S2 for an overview; duration: ∼20 min). Example materials for the scaffolding sessions can be found on OSF (https://osf.io/zac9d/). Learning facilitators logged notes for each session, and all sessions were recorded with permission from caregivers and children. To ensure fidelity of scaffolding implementation, select sessions from each learning facilitator were evaluated by a senior researcher on the team (a speech and language pathologist who helped design the intervention and trained the learning facilitators). The senior researcher watched the session videos and provided feedback as needed to help learning facilitators support participant interaction, adapt the scaffolding session scripts appropriately, and deliver the lessons as intended.

Mindfulness: Each participant in the Mindfulness condition completed mindfulness exercises provided by *Inner Explorer*. Participants were instructed to complete five 10‐min mindfulness practices per week for the intervention period. The efficacy of the mindfulness intervention was analyzed separately (see Treves, Li, et al. 2023, Treves, Olson, et al. 2023 for details). For these analyses, the Mindfulness condition served as the active control, as they did not receive any reading‐ or language‐based support.

### 2.5 Testing

A variety of measures were collected at the beginning (pre‐test) and end (post‐test) of the intervention period. The measures pertaining to the current paper are described below; information on additional measures can be found in Table S3.

Pre‐test: Each participant completed two or three pre‐test sessions, depending on how long it took them to complete the assessment battery. All assessments were conducted by experimenters blind to group assignment (researchers trained to administer psychoeducational assessments, independent from the learning facilitators) via Zoom. Assessments were administered in a priority order, with listening comprehension, nonverbal reasoning, and vocabulary measures administered during Pre‐test 1, and other language and reading measures administered during Pre‐test 2. Pre‐test 3 was completed after the first book was read (in the two text‐supplemented audiobooks groups; this allowed us to collect data on proximal measures pertaining to the first book), or approximately 2 weeks into the intervention period (for the Mindfulness group). The final book pre‐test measures were administered during Pre‐test 3, as well as any remaining measures that were not completed during the initial pre‐test sessions.

Post‐test: Post‐testing was completed in two or three sessions, conducted via Zoom by experimenters blind to group assignment (until the final questionnaire). Assessments were again administered in a priority order, with final book measures administered during the first post‐test session.

### 2.6 Outcome Measures

Standardized Vocabulary Assessments: Because we were interested in the effects of the text‐supplemented audiobook intervention on children's vocabulary, we administered two well‐established vocabulary measures to assess both receptive and expressive vocabulary: the Peabody Picture Vocabulary Test (PPVT‐5; Dunn 2018) was used to measure receptive vocabulary, and the vocabulary subtest of the Wechsler Abbreviated Scale of Intelligence (WASI‐II; Wechsler 2011) was used to measure expressive vocabulary. Each assessment was administered once during pre‐test and once during post‐test, using different versions. For a detailed description of these and all other measures, including reliability coefficients, see Table S3.

Proximal Vocabulary Assessments: In addition to utilizing the standardized assessments of vocabulary, we also developed book‐specific “proximal measures” of vocabulary. Given the brief nature of the intervention, it was possible that gains made during the intervention period would not be measured by the standardized assessments, which included words that may not have been encountered in the text‐supplemented audiobooks. Engaging with text‐supplemented audiobooks may boost vocabulary learning as we hypothesized, but if the words children learned from the books did not appear on the standard assessments, we would not be able to measure these gains. Thus, we developed proximal vocabulary tests for each of the 26 titles. For each book, we used frequency (how many times each word appears in the book), age of acquisition (Brysbaert and Biemiller 2017; Kuperman et al. 2012), and concreteness ratings (Brysbaert et al. 2014) to select 16 target words per book, as well as 4 “easy” words from the book matched on frequency and 4 “non‐book” words matched on age of acquisition that did not appear in the book. The target words were selected to be unfamiliar but useful in broader contexts, also known as “Tier 2” words in Beck et al.’s framework (Beck et al. 2002). We ensured that items did not repeat on books within the same track. To create each book‐specific test, we selected all words in the book within an age of acquisition bin based on the study‐specific book track (6–8 years for Track 1, 8–10 years for Track 2, and 10–12 years for Track 3). Next, we sorted all the words from high to low on frequency within the book, then selected words that would be appropriate for receptive items (based on concreteness ratings and experimenter judgment) and expressive items (based on experimenter judgment). These word lists, plus the page numbers in the book for each word, were provided to learning facilitators and used for the vocabulary instructional component of scaffolding sessions (as described in the Intervention section above). Along with the strategies described, learning facilitators could use photos to help explain the meaning of words, but not the illustrations used in the proximal vocabulary assessment.

**FIGURE 2:**
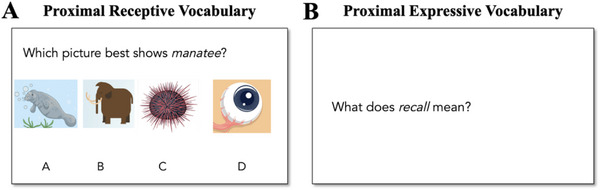
Example items from the Proximal Vocabulary Test for the book *Crenshaw*. (A) Receptive item. Options include the target word (A; manatee), a phonological foil (B; mammoth), a semantic foil (C; sea urchin), and an age‐of‐acquisition‐matched word (D; iris). (B) Expressive item. Items were read aloud to the child and displayed on the screen.

Each proximal vocabulary assessment contained receptive items (4‐choice multiple choice with pictures, analogous to the PPVT; Figure 2A) and expressive items (requiring students to define a word, scoring procedures based on the WASI‐2 vocabulary subtest; Figure 2B). We refer to these as the “Proximal Receptive Vocabulary” and “Proximal Expressive Vocabulary” tests, though it is important to note that these tests differ on a few dimensions. First, words with higher concreteness ratings were selected for the receptive vocabulary items so that we could represent them pictorially; in turn, the proximal expressive tests had lower overall concreteness ratings. Second, as is the case with the standardized assessments of receptive and expressive vocabulary we used in this study (PPVT and WASI‐2 vocabulary, respectively), the cognitive demands also differed between the tasks, with the expressive measure requiring children to draw on linguistic and metalinguistic skills to formulate a definition of the target word.

#### 2.6.1 Proximal Receptive Vocabulary Test

For the receptive items, we included one semantic foil and one phonological foil, plus an additional word matched on age of acquisition. The Proximal Receptive Vocabulary score is the sum of the number of correct responses to the 10 target words (maximum score = 10).

#### 2.6.2 Proximal Expressive Vocabulary Test

For the expressive items, participants were asked to define the target word. Responses were scored from 0 to 2 using a rubric, such that 0 = No Knowledge (e.g., no response, inappropriate use in phrase/sentence, inappropriate definition, restatement, phonological manipulation), 1 = Incomplete Knowledge (e.g., appropriate use in phrase/sentence [uses it without really defining the word], vague/imprecise definition, imprecise synonym, or 1‐H: homophone is defined rather than the target word), and 2 = Complete Knowledge (e.g., precise use in phrase/sentence, precise definition/synonym). Invalid responses (e.g., if upon scoring we noticed that a child looked up a definition online during the assessment) were scored as a 0. Independent experimenters used a subset of participant responses to create rubrics for each word in each book, with examples of what would constitute a 0, 1, and 2 response. Independent experimenters then scored a subset of new tests to measure reliability, and new experimenters scored the remaining responses. There were six target words per book (maximum score = 12).

#### 2.6.3 Administration

In order to obtain a pre‐test measure at the beginning of the intervention period, we administered a test based on the anticipated final book the child would complete, and we aimed to have each child in the Audiobooks‐Only and Audiobooks+Scaffold group read the intended book close to the end of the 8‐week intervention period. The tests were the same for pre‐test and post‐test for each book. In some cases, children in the Audiobooks‐Only and Audiobooks+Scaffold conditions did not read the intended final books and were instead post‐tested on the final book they completed. Tests were administered before and after each book for the Audiobooks+Scaffold group, so if they read a different final book, then they were pre‐tested during a scaffolding session prior to reading the final book (meaning that in these select cases, the test administrator was not blind to group assignment). The Mindfulness group did not read the books, so we picked the tests based on what would have been their assigned track based on their listening comprehension and receptive vocabulary scores.

### 2.7 Additional Measures

The following measures were not used as outcome variables but are relevant to other design components of the current paper.

#### 2.7.1 Listening Comprehension

Listening comprehension was assessed via the Understanding Spoken Paragraphs subtest of the Clinical Evaluation of Language Fundamentals (CELF‐5; Wiig et al. 2013). Listening comprehension was used to assign children to an appropriately‐leveled book track, and was also measured at post‐test.

#### 2.7.2 Nonverbal Reasoning

The matrices subtest of the Kaufman Brief Intelligence Test‐2 (KBIT‐2; Kaufman and Kaufman 2004) was administered during the first pre‐test session to measure nonverbal IQ and was used as an inclusion criterion for the study (standard score ≥80 on KBIT matrices; note that one participant was included with a lower KBIT score due to an initial scoring error).

#### 2.7.3 Audiobook Exposure

We measured audiobook exposure for the two text‐supplemented audiobook groups as the total number of minutes a child listened to recommended books. These data were automatically collected by the *Learning Ally* platform each time the child logged in, for each book the child listened to. *Learning Ally* provided these data to the study team.

#### 2.7.4 Reading Status

The DIBELS Passage Reading Fluency subtest was administered at pre‐test and was used to define Good Readers (>20th percentile) and Poor Readers (≤20th percentile) in our analyses (Good and Kaminski 2002).

#### 2.7.5 Socioeconomic Status (SES)

Parents filled out surveys about their child's background and home learning environments. Education levels were recorded for two primary caregivers, with the education levels being categorized into “Less than 7th Grade”, “Less than 9th Grade”, “Partial High School”, “GED”, “High School”, “Associate's degree”, “Bachelor's degree”, “Master's degree”, and “Doctorate”. These categories were transformed to 6, 8, 10, 12, 14, 16, 18, and 21 years of education, respectively, for subsequent analyses. SES was indexed by parental education and was calculated by averaging the number of years of education of both caregivers (if two were reported). Then, a median split was conducted on this average, with the median score set at 16 years. Participants were categorized as lower‐SES if their average fell below 16 years and higher‐SES if it was 16 years or above.

### 2.8 Missing Data

Missing values were present in pre‐test and post‐test measures, with the extent of missingness ranging from 0.32% to 33.44% (see Table S7 for missingness for each measure). To evaluate the nature of this missing data, we conducted Little's Missing‐Completely‐At‐Random (MCAR) test using the “naniar” package in R. Little's MCAR test was not significant (*χ* ^2^ = 1946, df = 4989, *p* >0.998), indicating that the missing data pattern was consistent with the MCAR assumption . A follow‐up attrition analysis revealed that while the missingness did not significantly differ by Reading Status (*t*(166.59) = 0.482, *p* = 0.651) or performance on the executive function measure, the BRIEF, (*t*(134.04) = 0.193, *p* = 0.847), but missingness was significantly greater in children from lower‐SES backgrounds (*t*(216.32) = 2.734, *p* = 0.007). This finding led us to cautiously proceed under the assumption that the data were Missing at Random (MAR). This assumption suggests that the likelihood of missing values is systematically related to observed variables, such as SES, but not to unobserved data. Therefore, we used multiple imputation by chained equations (mice; predictive mean matching) with ***m* = 20** imputations and **30 iterations**, including all analysis variables. Models were fit in each imputed dataset and **pooled via Rubin's rules**. Effect sizes were computed after pooling.

### 2.9 Data Analysis

To evaluate the main effects of the intervention on each primary outcome variable, we conducted ANCOVA models with multiple imputation (*m* = 20) using the *mice* package in R. Each model examined post‐test vocabulary as a function of intervention group (Audiobooks‐Only, Audiobooks+Scaffold, Mindfulness) while controlling for pre‐test scores, age, gender, parental education (lower/higher), nonverbal IQ (KBIT matrices), and baseline reading status (good/poor reader, based on DIBELS PRF percentile) (see Table S5 for models including book track, test interval for proximal measures, and whether the same final book was completed across pre‐ and post‐tests). Model estimates were pooled across imputations using *mice::pool()*. Effect sizes (Cohen's *d*) were computed from the pooled model estimates by dividing intervention coefficients (Audiobooks‐Only, Audiobooks+Scaffold) by the pooled post‐test standard deviation within the Mindfulness control group, expressing effects relative to control‐group variability.

To assess whether intervention effects varied by baseline reading status or SES, we conducted moderation analyses using the same model specification (Dunnett 1955; Rubin 1987). Reading status was defined by DIBELS PRF (≤20th percentile = poor reader), and SES was defined by average parental education (<16 years = lower SES). Within each subgroup (e.g., good vs. poor readers; higher vs. lower SES), we fit multiply imputed ANCOVA models predicting post‐test scores from pre‐test scores, age, gender, nonverbal IQ, and relevant covariates (reading status and SES). To formally test for moderation, we also fit ANCOVA models including the Moderator x Group interaction term (with Mindfulness as reference). A parallel two‐group model (Audiobook‐Only as reference) tested the Scaffold vs. Audiobooks‐Only interaction.

Estimated marginal means (EMMs) for each intervention group were obtained using the *emmeans* package, and pairwise contrasts comparing each intervention to the Mindfulness control group were tested using Dunnett's method (Dunnett 1955). Contrast estimates, standard errors, and test statistics were pooled across imputations using Rubin's rules (Rubin 1987). Effect sizes (Cohen's *d*) were calculated by dividing the pooled contrast estimate by the pooled control‐group standard deviation within each subgroup, allowing for consistent interpretation across models while accounting for uncertainty due to missing data.

Finally, we examined whether audiobook exposure (total minutes listened) differed between the two audiobook groups (Audiobooks‐Only vs. Audiobooks+Scaffold) using multiply imputed linear models with group as the sole predictor.

The overall analysis plan for this study was preregistered before data analysis; see https://osf.io/ucgvj (and see Supporting Information for notes on deviations from the preregistration).

## 3 Results

### 3.1 Baseline Measures

At pretest, participants in the three groups did not differ in standardized measures of vocabulary and passage reading fluency. For receptive vocabulary (PPVT), the mean raw score was 172 (*SD* = 17.7) in the Audiobooks‐Only group, 169 (*SD* = 21.7) in the Audiobooks+Scaffold group, and 172 (*SD* = 20.2) in the Mindfulness group. For expressive vocabulary (WASI), means were 27.7 (*SD* = 5.45) in the Audiobooks‐Only group, 27.0 (*SD* = 5.80) in the Audiobooks+Scaffold group, and 27.4 (*SD* = 5.25) in the Mindfulness group. For passage reading fluency (DIBELS PRF percentile score), means were 47.5 (*SD* = 33.5) in the Audiobooks‐Only group, 48.4 (*SD* = 30.7) in the Audiobooks+Scaffold group, and 47.8 (*SD* = 33.8) in the Mindfulness group. ANOVA tests revealed no significant differences between groups on the PPVT raw scores (*F*(2, 311) = 0.70, *p* = 0.496), WASI raw scores (*F*(2, 311) = 0.40, *p* = 0.669), or the DIBELS PRF percentile scores (*F*(2, 311) = 0.02, *p* = 0.978), indicating that groups were comparable at baseline. There were also no group differences in age, race/ethnicity, nonverbal reasoning (KBIT matrices), reading fluency, proportion of poor readers, grade level, schooling experience during the study, or parental education (see Table 1). Attrition was distributed across groups without indication of imbalance or bias.

### 3.2 Main Effects on Receptive and Expressive Vocabulary

For each measure, we included pre‐test score, age, gender, SES (lower/higher parental education), reading status (good/poor reader based on DIBELS PRF score cutoffs), nonverbal IQ, and intervention group terms in the model (Table 2). See Table S4 for full models.

**TABLE 2:** Main outcome measures.

Measure	Group	Est.	S.E.	t‐value	*p* value	Effect size
Proximal Receptive Vocabulary	Audio > Mind	0.238	0.250	0.95	0.342	0.15
	Scaffold > Mind	1.010	0.252	4.01	**<0.001**	0.64
Proximal Expressive Vocabulary	Audio > Mind	0.764	0.304	2.51	**0.013**	0.35
	Scaffold > Mind	1.636	0.314	5.21	**<0.001**	0.74
Standard Receptive Vocabulary (PPVT)	Audio > Mind	−1.541	2.113	−0.73	0.467	−0.10
	Scaffold > Mind	−0.888	2.078	−0.43	0.669	−0.06
Standard Expressive Vocabulary (WASI)	Audio > Mind	−0.133	0.566	−0.23	0.815	−0.02
	Scaffold > Mind	−0.353	0.553	−0.64	0.523	−0.07

*Note*: Audio = Audiobooks‐Only; Scaffold = Audiobooks+Scaffold; Mind = Mindfulness (control group).

**FIGURE 3:**
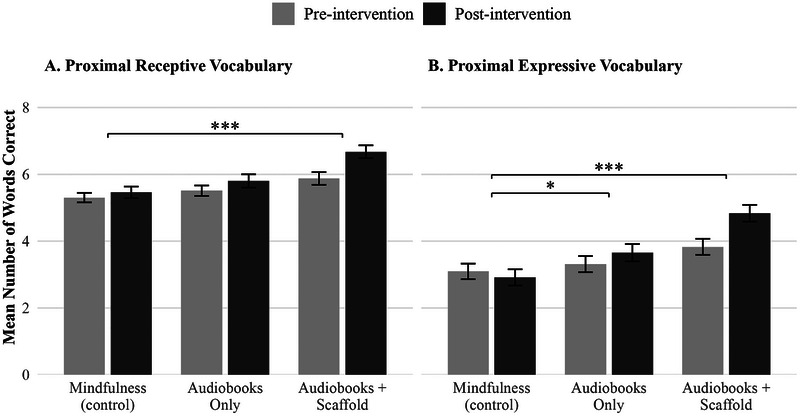
Summary of intervention effects for proximal vocabulary measures. Mean raw scores (number of words correct) on the proximal receptive vocabulary and expressive vocabulary measures at pre‐test (light gray) and post‐test (dark gray) measurement occasions by intervention group. Brackets indicate contrasts computed using estimated marginal means with Dunnett's method (treatment vs. control) and pooled across *m* = 20 imputations using Rubin's rules. Error bars represent ±1 SE. **p < 0.001, *p < 0.01, *p* < 0.05, †*p* < 0.10.

The most likely measures to capture changes in children's language over the course of the 8‐week intervention were the proximal measures of receptive and expressive vocabulary. These two measures were designed to assess children's knowledge of words that appeared in the texts and were taught in scaffold sessions; thus, if children learned these words through listening to the books or participating in scaffolding sessions, their scores should improve. Importantly, children's scores on the proximal vocabulary measures were correlated with their scores on the standard vocabulary measures at pre‐test, suggesting that the proximal vocabulary measures captured meaningful variation in children's vocabulary skills (Figure S1). Children in the Audiobooks+Scaffold group showed a significant improvement in proximal receptive vocabulary compared to the control group, but not children in the Audiobooks‐Only group (Table 2). However, children in both the Audiobooks‐Only and the Audiobooks+Scaffold group showed significant improvements in proximal expressive vocabulary compared to the control group, with the effect size being almost twice as large for the Audiobooks+Scaffold compared to Audiobooks‐Only (Table 2). Figure 3 shows proximal vocabulary scores at pre‐test and post‐test by group; scores are significantly higher at post‐test compared to pre‐test for the Audiobooks+Scaffold group (for both expressive and receptive vocabulary) and the Audiobooks‐Only group (for proximal expressive vocabulary), but not for the control group (Mindfulness).

Next, we examined effects on standardized measures of receptive and expressive vocabulary. Unlike the proximal measures, there was no effect of group assignment for either receptive or expressive vocabulary using standardized measures (Table 2).

### 3.3 Moderating Effects on Proximal Vocabulary Outcome Measures

We examined whether treatment effects were moderated by reading status (good vs. poor readers) or socioeconomic status (higher vs. lower parental education) using ANCOVA models with *Group × Moderator* interactions. Models included Mindfulness (control group) as the reference group, allowing us to test whether each intervention showed differential effectiveness across subgroups. We first examined reading status and SES as potential moderating factors of proximal vocabulary gains.

**FIGURE 4:**
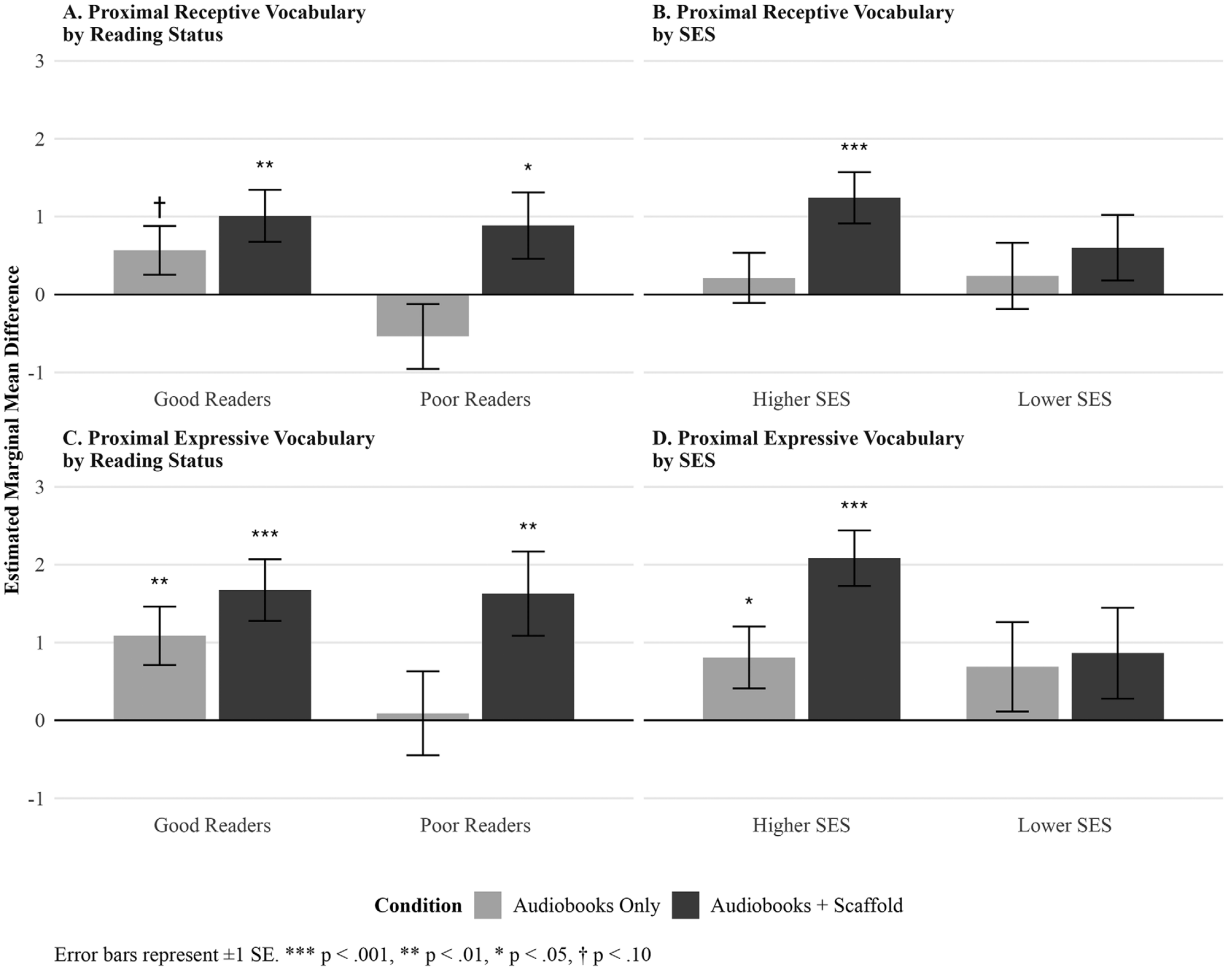
Effects of intervention on vocabulary pretest to posttest gains by reading status and SES. Effects were derived from ANCOVA models with estimated marginal mean contrasts with Dunnett's adjustment and pooled across *m* = 20 imputations. Audiobooks‐Only group in light gray; Audiobooks+Scaffold group in dark gray. Error bars: ±1 SE. †*p* < 0.1; *p < 0.05; **p < 0.01; ***p < 0.001.

For proximal receptive vocabulary, there was a marginally significant interaction between reading status and the Audiobooks‐Only > Mindfulness contrast (*b* = −1.049, *SE* = 0.536, *p* = 0.052), but not between reading status and the Audiobooks+Scaffold > Mindfulness contrast (*b* = −0.120, *SE* = 0.563, *p* = 0.832). The difference in interaction coefficients between Audiobooks+Scaffold and Audiobooks‐Only conditions (*b* = 0.949, *SE* = 0.553, *p* = 0.089) indicates that poor readers benefited marginally more from scaffolding relative to audiobooks alone compared to good readers. Numerically, poor readers did not benefit from audiobooks alone (*d* = ‐0.35) but showed significant gains from scaffolding (*d* = 0.57), with a large scaffolding advantage over audiobooks alone (*d* = 0.91). Good readers showed benefits with moderate effect for both interventions (Audiobooks‐Only, *d* = 0.35; Audiobooks + Scaffold, *d* = 0.63; see Figure 4A). For SES, there was no significant interaction with the Audiobooks‐Only > Mindfulness contrast (*b* = 0.032, *SE* = 0.528, *p* = 0.951) or the Audiobooks+Scaffold > Mindfulness contrast (*b* = −0.596, *SE* = 0.560, *p* = 0.290).

For proximal expressive vocabulary, the interaction between reading status and the Audiobooks‐Only > Mindfulness contrast was not significant (*b* = −0.922, *SE* = 0.704, *p* = 0.194), nor was the interaction with the Audiobooks+Scaffold > Mindfulness contrast (*b* = −0.007, *SE* = 0.677, *p* = 0.992). The difference in interaction coefficients was also not significant (*b* = 0.942, *SE* = 0.680, *p* = 0.169), though suggests a similar pattern to receptive vocabulary, with poor readers showing numerically greater gains from scaffolding compared to audiobooks alone (Audiobooks‐Only, *d* = 0.04; Audiobooks + Scaffold, *d* = 0.69; see Figure 4C). For SES, the interaction with the Audiobooks‐Only > Mindfulness contrast was not significant (*b* = −0.114, *SE* = 0.716, *p* = 0.874), while the interaction with the Audiobooks+Scaffold > Mindfulness contrast approached significance (*b* = −1.169, *SE* = 0.652, *p* = 0.076). The difference between these interaction terms was not significant (*b* = −1.074, *SE* = 0.689, *p* = 0.124). Descriptively, higher‐SES children showed large benefits from scaffolding (*d* = 0.99) compared to more modest benefit from audiobooks alone (*d* = 0.39) whereas lower‐SES children showed modest, similar benefits from both interventions (Audiobooks+Scaffold, *d* = 0.37, vs. Audiobooks‐Only, *d* = 0.29; see Figure 4D.

Exploratory subgroup analyses (Figure 4; Table 3) revealed that, after controlling for SES, good readers showed improvements in both the Audiobooks+Scaffold and Audiobooks‐Only groups, whereas poor readers improved only in the Audiobooks+Scaffold group for both proximal measures. After controlling for baseline reading ability, higher‐SES students showed significant gains in both Audiobooks‐Only and Audiobooks+Scaffold conditions for expressive vocabulary (and in the Audiobooks+Scaffold condition for receptive vocabulary), whereas lower‐SES students did not show significant benefits in either condition.

**TABLE 3:** Summary of intervention effects for proximal measures.

Proximal receptive vocabulary						
Group	Contrast	Est.	S.E.	t‐value	*p* value	Effect size
**Good Readers**	Audio > Mind	0.566	0.312	1.815	0.071	0.35
	Scaffold > Mind	1.009	0.334	3.019	**0.003**	0.63
	Scaffold > Audio	0.443	0.289	1.534	0.125	0.28
**Poor Readers**	Audio > Mind	−0.539	0.417	−1.292	0.197	−0.35
	Scaffold > Mind	0.885	0.425	2.081	**0.038**	0.57
	Scaffold > Audio	1.424	0.452	3.151	**0.002**	0.91
**Higher SES**	Audio > Mind	0.214	0.321	0.665	0.507	0.13
	Scaffold > Mind	1.241	0.329	3.767	**<0.001**	0.76
	Scaffold > Audio	1.028	0.307	3.350	**<0.001**	0.63
**Lower SES**	Audio > Mind	0.239	0.424	0.563	0.574	0.16
	Scaffold > Mind	0.601	0.420	1.433	0.154	0.40
	Scaffold > Audio	0.362	0.421	0.860	0.390	0.24

**Proximal expressive vocabulary**						
**Group**	Contrast	**Est**.	**S.E**.	**t‐value**	***p* value**	**Effect size**
**Good Readers**	Audio > Mind	1.086	0.376	2.889	**0.005**	0.52
	Scaffold > Mind	1.674	0.396	4.224	**<0.001**	0.80
	Scaffold > Audio	0.588	0.347	1.695	0.091	0.28
**Poor Readers**	Audio > Mind	0.091	0.538	0.169	0.866	0.04
	Scaffold > Mind	1.628	0.541	3.006	**0.003**	0.69
	Scaffold > Audio	1.537	0.557	2.760	**0.006**	0.65
**Higher SES**	Audio > Mind	0.808	0.398	2.030	**0.046**	0.39
	Scaffold > Mind	2.084	0.357	5.840	**<0.001**	0.99
	Scaffold > Audio	1.276	0.371	3.436	**<0.001**	0.61
**Lower SES**	Audio > Mind	0.687	0.575	1.196	0.239	0.29
	Scaffold > Mind	0.862	0.584	1.477	0.150	0.37
	Scaffold > Audio	0.175	0.526	0.333	0.740	0.08

*Note*: Intervention effects within subgroups defined by reading status and SES. Effects were derived from ANCOVA models using estimated marginal mean contrasts with Dunnett's adjustment for treatment‐versus‐control comparisons, pooled across m = 20 imputations. Effect sizes (Cohen's d) were calculated using Mindfulness group standard deviation within each subgroup. Audio = Audiobooks‐Only; Scaffold = Audiobooks+Scaffold; Mind = Mindfulness (control group).

**FIGURE 5:**
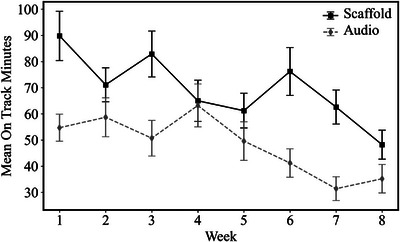
Week‐by‐week total number of minutes spent listening to recommended text‐supplemented audiobooks for the Audiobooks+Scaffold (black) and Audiobooks‐Only (hashed gray) groups.

There was a significant difference in audiobook exposure, with children in the Audiobooks+Scaffold group listening to recommended audiobooks for more minutes in total (*M*(*SD*) = 556.9(423.06) min) than children in the Audiobooks‐Only group (*M*(*SD*) = 384.8(335.88) min) during the 8‐week intervention period (*b* = 168.49, *SE* = 56.01, *t*(422) = 3.008, *p* = 0.003; Figure 5). Week‐by‐week audiobook exposure data revealed higher audiobook exposure in the Audiobooks+Scaffold group than the Audiobooks‐Only group in Week 1 (*b* = 35.08, *SE* = 10.7, *t*(826) = 3.289, *p* = 0.001), Week 3 (*b* = 32.20, *SE* = 10.7, *t*(826) = 3.019, *p* = 0.003), Week 6 (*b* = 34.99, *SE* = 10.7, *t*(826) = 3.281, *p* = 0.001), and Week 7 (*b* = 31.21, *SE* = 10.7, *t*(826) = 2.926, *p* = 0.004). However, there was no significant difference in audiobook exposure for Week 2 (*b* = 12.32, *SE* = 10.7, *t*(826) = 1.332, *p* = 0.249), Week 4 (*b* = 1.79, *SE* = 10.7, *t*(826) = 0.168, *p* = 0.866), Week 5 (*b* = 11.62, *SE* = 10.7, *t*(826) = 1.089, *p* = 0.276), or Week 8 (*b* = 12.93, *SE* = 8.81, *t*(826) = 1.212, *p* = 0.226).

Because audiobook exposure differed between these conditions (Figure 5), we tested whether the total minutes spent listening to recommended audiobooks moderated the association between group assignment and intervention response on the proximal vocabulary measures. Listening time significantly moderated intervention effects for expressive vocabulary (*b* = 0.001, *SE* = 0.001, *t* = 1.97, *p* = 0.049) but not receptive vocabulary (*b* = 0.000, *SE* = 0.001, *t* = 0.221, *p* = 0.825).

## 4 Discussion

We implemented a fully remote, RCT intervention to test whether listening to audiobooks paired with text, with and without one‐on‐one scaffolded support, would improve children's vocabulary skills relative to a mindfulness condition without exposure to audiobooks. Indeed, both audiobook intervention groups improved on proximal measures of expressive vocabulary from pre‐test to post‐test, and the Audiobooks+Scaffold group showed improvements in receptive vocabulary as well. Therefore, children learned new vocabulary both incidentally through exposure (in the context of reading and listening) as well as through explicit instruction. The effect sizes we observed in the Audiobooks+Scaffold group (*d* = 0.64 for proximal receptive vocabulary and *d* = 0.74 for proximal expressive vocabulary) were comparable to previous literature on read‐aloud interventions in classroom settings that included teacher‐read materials followed by explicit instruction of vocabulary (*g* = 0.53 in a meta‐analysis by Cervetti et al. 2023, and *d* = 0.57–1.71 in Fien et al. 2011 and Loftus and Coyne 2013).

Previous vocabulary intervention studies have shown that brief, explicit instruction can yield measurable gains for students with low baseline vocabularies, even over an eight‐week period (Fien et al. 2011; Loftus and Coyne 2013). In our study, students received explicit instruction on approximately 32 target words across several books in the Audiobooks+Scaffold group, but because learning was assessed only on the final book, they appeared to have mastered one to two of the few words taught in that section. Assuming a similar rate of retention across the 32 taught words, this corresponds to roughly 15–30 percent of instructed vocabulary, which, if sustained across a typical 36‐week school year, would translate to approximately 150–300 new words learned annually and 1800–3600 words across K–12—consistent with long‐standing estimates that typical students acquire about 2000 to 4000 words through explicit instruction out of roughly 40,000 to 50,000 total word families by high school graduation (Beck and McKeown 2007; Biemiller and Slonim 2001; Nagy and Anderson 1984; Stahl and Nagy 2007).

Importantly, the benefits experienced by the intervention groups were influenced by the students' baseline reading abilities. Good readers (as defined based on their reading fluency) showed enhancements across both text‐supplemented audiobook conditions, whereas poor readers exhibited significant vocabulary gains only within the Audiobooks+Scaffold condition. SES, operationalized by parental education, also moderated the intervention effects: higher‐SES students benefited more from scaffolded support, while lower‐SES students showed only marginal gains in both conditions. Finally, children who received scaffolded support listened to audiobooks for more time than children in the Audiobooks‐Only group, which moderated intervention response for expressive vocabulary. Together, these results suggest a promising potential role for audiobooks in combination with individualized explicit and systematic instruction for improving skills important for reading comprehension, such as vocabulary knowledge.

### 4.1 Efficacy of Voluntary Listening to Text‐Supplemented Audiobooks

Our findings indicate that children who were granted access to a carefully curated text‐supplemented audiobook library incidentally learned new words and improved their expressive vocabulary knowledge. This aligns with substantial evidence that children learn vocabulary through independent reading (Cunningham and Stanovich 1991; Nagy et al. 1985, Nagy et al. 1987), and with evidence that orally presented reading materials can enhance language skills (Beck and McKeown 2001). Reading aloud texts that are beyond a child's independent reading level is a recommended educational practice (Shanahan et al. 2010). However, such read‐alouds are considerably time‐consuming for teachers, especially with longer materials in higher grades, leading to limited implementation of this practice (Hoffman et al. 1993; Merga and Ledger 2019). Moreover, tailoring linguistically challenging materials to create the “just right” level for each child is not always feasible in a whole‐classroom instructional context with high variability in children's language and content knowledge. When the read‐aloud instruction is whole‐class based and not individualized, vocabulary gains are significantly related to children's baseline differences in language (Coyne et al. 2007, 2009, 2019; Penno et al. 2002). Thus, it remains uncertain whether teacher‐facilitated oral reading can effectively bridge the gaps in vocabulary knowledge between proficient and struggling readers, which partially emerge via differences in their independent reading habits (Biemiller 2004; Coyne et al. 2007, 2019).

Alternative approaches to mitigating knowledge and reading skill gaps have aimed to boost reading engagement by promoting independent reading. Unfortunately, evidence for the effectiveness of these approaches is weak and indicates that these strategies have limited effects on enhancing language skills (Erbeli and Rice 2022). Our study presents a novel hybrid approach, effectively combining the benefits of read‐alouds—implemented independently through audiobooks—and the promotion of independent reading. This was achieved by providing access to the *Learning Ally* platform, curating a selection of text‐supplemented audiobooks suitable for different reading levels, and sending regular reminders to encourage reading. Although we could not isolate the extent to which students relied on the orally presented versus written content of the text‐supplemented audiobooks, our results are encouraging in that children improved their expressive vocabulary skills through incidental learning in the Audiobooks‐Only group compared to the Mindfulness control group. This suggests that providing access to high‐quality and appropriately challenging audiobooks may serve as a scalable child‐facilitated alternative to a read‐aloud intervention.

### 4.2 Effects of Explicit and Systematic One‐On‐One Instruction

In the Audiobooks+Scaffold condition, along with providing text‐supplemented audiobook access, we delivered an explicit and individualized intervention for children twice a week for eight weeks. In accordance with the best practices recommended for enhancing reading comprehension skills (Shanahan et al. 2010; Wright and Cervetti 2017), our approach was aimed at supporting both the breadth and depth of children's vocabulary learning. This was achieved by presenting multiple meanings and usages, developing morphological awareness, and encouraging the use of words in meaningful contexts (Beck et al. 1982; Language and Reading Research Consortium (LARRC), Arthur, et al. 2016). Additionally, our intervention involved teaching children specific comprehension strategies such as making inferences, understanding narrative and expository texts, and applying comprehension monitoring, all of which are crucial for developing active and engaged readers who can construct meaning from texts (Cain et al. 2001, 2004; Clarke et al. 2010). Because we used an existing evidence‐based curriculum, we did not experimentally manipulate or measure the relative contributions of the different curricular components (e.g., vocabulary instruction vs. comprehension strategies), although this could be an area for future research.

Vocabulary improvements in the Audiobooks+Scaffold group are consistent with previous studies that have implemented read‐aloud interventions paired with teacher‐directed explicit small‐group comprehension and vocabulary instruction (D. L. Baker et al. 2020; S. K. Baker et al. 2013; Fien et al. 2011, cf., Beck et al. 1982; Clarke et al. 2010). Students typically learn only a small fraction of unfamiliar words they encounter during independent reading (Swanborn and de Glopper 1999); thus, it makes sense that the Audiobooks+Scaffold group showed larger gains on vocabulary measures than the Audiobooks‐Only group. The intervention implemented in this study was adapted from a previously validated whole‐class full‐year curriculum (Language and Reading Research Consortium (LARRC) et al. 2019), which showed a positive impact on proximal vocabulary knowledge and distal effects on reading comprehension skills (in Grade 3, but not 1 or 2).

### 4.3 Improvements on Both Expressive and Receptive Vocabulary Measures

It is notable that students in the Audiobooks+Scaffold group improved across measures of both expressive and receptive vocabulary, which differed in a number of ways. The proximal receptive vocabulary measure assessed children's ability to recognize the meaning of a word, whereas the proximal expressive vocabulary measure required children to provide a definition—thus, the expressive vocabulary measure required a deeper and more precise understanding of the target word and ability to express its meaning (i.e., drawing on both linguistic and metalinguistic demands to formulate an appropriate definition). For both measures, the Audiobooks+Scaffold group made larger gains than the Audiobooks‐Only group, suggesting an added benefit of the scaffolding sessions for vocabulary learning. Indeed, the scaffolding sessions in the present study included instruction of words included in both the receptive and expressive proximal vocabulary assessments, using evidence‐based strategies including practicing word pronunciations, building morphological awareness, discussing multiple meanings of the word, and using the word in different contexts. Most previous studies either did not assess both measures or failed to identify differences in their effects. Importantly, vocabulary knowledge is not binary but rather varies across a spectrum (e.g., Dale 1965). Because the scoring system for the expressive vocabulary measure gave partial credit for approximate definitions, the greater improvements in the Audiobooks+Scaffold group could also indicate that this group learned more specific representations of targeted vocabulary words.

### 4.4 Intervention Effects Were Moderated by Reading Ability and SES

In this study, good readers (i.e., those who scored above the 20^th^ percentile on passage reading fluency) improved from pre‐test to post‐test on the proximal expressive vocabulary measure regardless of whether they were in the Audiobooks‐Only group or the Audiobooks+Scaffold group, and they improved on the proximal receptive vocabulary measure in the Audiobooks+Scaffold group. For both measures, there was no additional benefit of being in the Audiobooks+Scaffold group. Critically, for poor readers, it was only the Audiobooks+Scaffold group that saw proximal vocabulary gains as a result of the intervention. Thus, simply removing decoding as a barrier to accessing vocabulary (through the use of text‐supplemented audiobooks) was insufficient. This is consistent with prior evidence that explicit and targeted instructional support may be especially important for struggling readers (Duff 2019; Rupley et al. 2009), including a prior study that likewise found that vocabulary learning from context was greater in more proficient readers (Jenkins et al. 1984). It is also consistent with prior work finding that independent reading interventions have little effect on student reading gains overall and in struggling readers specifically (Erbeli and Rice 2022; Kim 2007).

In this study, we used a text decoding measure (passage reading fluency) to define our “poor reader” group, but children can struggle with reading due to many (often overlapping) reasons (Carretti et al. 2009; Catts et al. 2006; McCardle et al. 2001; Stothard and Hulme 1992). Drawing on the “simple view of reading” framework (Gough and Tunmer 1986; Hoover and Gough 1990), which highlights the separate components of decoding ability and language comprehension that contribute to reading comprehension, the audiobook intervention components can be conceptualized as targeting separate components of reading: text‐supplemented audiobooks support decoding, while the scaffolding sessions support language comprehension. While we identified our “poor reader” group based on a text decoding measure, many students may have struggled with reading due to multiple factors beyond decoding. Indeed, poor readers did not significantly improve when only decoding challenges were mitigated (i.e., in the Audiobook‐Only group).

Vocabulary learning was also influenced by SES. In the higher SES group, there were stronger gains in vocabulary in the Audiobooks+Scaffold condition than the Audiobooks‐Only condition across both expressive and receptive proximal vocabulary measures. The lower SES group, however, did not show statistically significant improvements in either vocabulary measure, and they did not benefit more from being in the Audiobooks+Scaffold condition compared to the Audiobooks‐Only condition. We were not the first to find stronger intervention effects amongst more advantaged students: a meta‐analysis of vocabulary interventions found that lower‐SES children made lower gains on word learning than higher‐SES children (Marulis and Neuman 2013), consistent with our results. In the present study, the “higher” and “lower” SES groups were based on a median split of parental education, which limits the inferences we can make about their experiences, especially when comparing to prior studies. Indeed, because our median split was at 16 years of average parental education, the “lower” SES group includes participants whose parents completed at least some post‐secondary education, which would not be considered low SES more generally.

Why did lower‐SES participants not show significant improvements on either of the proximal vocabulary measures in the present study, especially in the Audiobooks+Scaffold group? Some summer reading interventions have been more effective for lower‐SES students compared to their higher‐SES peers (Kim and Quinn 2013; Romeo et al. 2018), suggesting that improving access and promoting independent reading may be effective. We expected that the Audiobooks+Scaffold condition to be especially effective by providing one‐on‐one support. For instance, in an e‐book intervention for low‐SES students, the combination of one‐on‐one support (joint reading with the mother) combined with explicit instruction (via an e‐book dictionary) improved vocabulary learning more than either of those supports on their own (Korat and Shneor 2019). Combining motivational support with reading strategy instruction was also an effective strategy in a previous study with low‐SES fifth graders (Ng et al. 2013). One explanation for the lack of gains in our study may have been that our relatively lower‐SES group was more advantaged than lower‐SES groups in prior studies. It is also possible that lower‐SES participants in our study dealt with factors that made the Audiobooks+Scaffold condition less effective, such as higher stress or lack of access to a quiet, focused space due to the pandemic context. Since the scaffold sessions focused on interactive dialogue about the books, which has been shown to improve expressive vocabulary in previous studies (Sénéchal 1997; Whitehurst et al. 1988), this could have led to comparable effects of the Audiobooks‐Only and Audiobooks+Scaffold groups specifically for expressive vocabulary.

Overall, relationships between low‐SES and reading difficulties are complex and multi‐faceted (Buckingham et al. 2013), and thus, intervention efficacy may differ depending on different underlying factors associated with lower‐SES and reading performance. While we cannot untangle the mechanism explaining the lack of vocabulary improvement for lower‐SES participants in this study without further research, an important takeaway is that the optimal strategy for using audiobooks to promote vocabulary growth may differ for lower‐SES students. These findings support the importance of taking student demographic factors into consideration when designing and assessing educational interventions and instructional content (e.g., teacher‐based factors; Haycock 2001; Koedel 2009; Sanders and Horn 1998).

### 4.5 Higher Audiobook Exposure With Text‐Supplemented Audiobooks in the Audiobooks+Scaffold Condition

In addition to vocabulary growth, children who received one‐on‐one scaffolded support during the intervention period also spent more time listening to the text‐supplemented audiobooks than children who only received text‐based reminders to listen to their books. Previous interventions aimed at boosting independent reading have shown limited success (Erbeli and Rice 2022; National Reading Panel [US] & National Institute of Child Health, & Human Development [US], 2000). It is possible that the social accountability of meeting to discuss the books, the enthusiasm of the learning facilitators, the implementation of comprehension strategies, or other factors contributed to this difference. If one‐on‐one scaffolded support results in long‐term boosts in independent reading, which in turn increases exposure to new content and vocabulary, this could be a potential mechanism for improving reading comprehension, as independent reading has been associated with better reading comprehension (Wantchekon and Kim 2019).

An important contributor to interventions focused on independent reading is student choice (Kim et al. 2021). Although children were constrained to a set of titles that were selected based on their listening comprehension skills, they could choose from a diverse set of options, including fiction and nonfiction books. Choice can motivate children to read more (Fisher and Frey 2018; Guthrie et al. 2007), and a large body of research has also shown that children perform better on reading comprehension assessments when the material is interesting or familiar (Baldwin et al. 1985; Kendeou et al. 2003; Recht and Leslie 1988; Shnayer 1968). Thus, while not empirically tested, it is possible that the element of student choice impacted the efficacy of the study given that both text‐supplemented audiobook groups spent time listening to the books and showed improvements in vocabulary knowledge. Indeed, the majority of caregivers surveyed at the end of the study—in both text‐supplemented audiobook groups—said that their child liked the recommended books “somewhat” or “a lot” (Ozernov‐Palchik et al. 2022).

### 4.6 Remote Study Context

An important context for interpreting the results of this remote study is that it began during the first few months of the Covid‐19 pandemic, during the summer of 2020, and continued throughout the height of the pandemic. Throughout the study, as evidenced by anecdotal reports and survey responses we collected, children and families navigated a mix of remote, hybrid, and in‐person learning as well as many personal challenges. Indeed, one motivation for beginning this study when we did was to specifically try to support children at higher risk for learning loss during school disruptions. Prior research suggests that students from lower‐SES backgrounds tend to have greater learning loss over summer vacations than their higher‐income peers (e.g., Cooper et al. 1996; cf., von Hippel et al. 2018), and that summer reading interventions may be particularly beneficial for low‐income children (Kim and Quinn 2013; Romeo et al. 2018). Children with reading difficulties also lose ground over summer breaks (Christodoulou et al. 2017). There is now substantial evidence that children experienced learning loss during the pandemic, with greater negative effects on children from disadvantaged environments (Donnelly and Patrinos 2022; Engzell et al. 2021; Goldhaber et al. 2022; Khan and Ahmed 2021). Furthermore, the pandemic greatly disrupted children's routines and social structures. Anecdotally, multiple caregivers in the Audiobooks+Scaffold group commented that the scaffolding sessions were one of the only stable one‐on‐one relationships their child had outside the household during this time period. Given this context, it is therefore possible that this intervention approach may have had larger—or smaller—effects if conducted during a less tumultuous time.

### 4.7 Translational Potential

Despite all the challenges posed by the pandemic, this study nonetheless had positive outcomes on children's vocabulary, suggesting robustness in the overall approach of pairing text‐supplemented audiobooks with scaffolding to promote vocabulary learning. Furthermore, it is *because* of the pandemic that this study necessitated an easily scalable design. All required study materials could be distributed remotely, including the *Learning Ally* platform. The scaffolding sessions were conducted by college students, who were trained and monitored by research staff via virtual tools and recordings. Even the assessments were administered online, allowing families to schedule at convenient times without requiring students to come into a lab or miss out on learning time at school. Not only do our results suggest that remote intervention studies are a promising approach to increasing participant reach (see Ozernov‐Palchik et al. 2022), but they also lay the foundation for specifically scaling up a learning strategy that incorporates both audiobooks and scaffolded one‐on‐one instructional support.

Remote learning is made possible by technology, and there is hope that education technology can enhance educational outcomes on behalf of the most vulnerable students: those with learning differences and those coming from disadvantaged environments. There is as yet, however, minimal evidence that such technology accomplishes this goal of educational equity. The present study indicates that vulnerable students may best be served by a blend of human support and technology. Indeed, the same conclusion was reached in a study of artificial intelligence (AI) math tutoring that found that combined human and technology‐based tutoring yielded best outcomes (Chine et al. 2022).

From a scalability standpoint, we found that minimal training sufficed for non‐professionals (i.e., college students) to effectively deliver a clear, evidence‐based curriculum paired with independent reading via text‐supplemented audiobooks. By using text‐supplemented audiobooks, children could engage with more challenging texts without requiring in‐person instruction time or caregiver involvement. The scaffolding sessions supplemented independent reading, requiring only brief one‐on‐one interactions at times convenient for families and implementable by minimally trained individuals. Essentially, our approach “unbundled” the reading and discussion components of read‐alouds, delivering both in a flexible, resource‐light manner outside the school setting. We see particular promise in scaling this further through the use of non‐professional volunteers or even artificial intelligence to tailor lessons without increasing demands on teacher time.

### 4.8 Limitations and Future Directions

While the remote study context provided numerous benefits, it also introduced a few limitations (see Ozernov‐Palchik et al. 2022 for discussion). As one might expect, there were some technical challenges and scheduling difficulties that led to certain tests being administered later than planned or missing altogether. Furthermore, because we succeeded in recruiting a diverse sample of children across the United States, participants experienced vastly different schooling circumstances during the study period (learning in person, learning remotely, hybrid learning, vacation, etc.). It is worth noting that the analyses in this paper did not take into account some factors that may have impacted intervention adherence or outcomes, though these concerns are mitigated by the RCT design.

Despite the promising effects of the intervention on book‐specific measures of proximal vocabulary, there were no significant effects on more distal measures of vocabulary or other language skills. Most studies examining the efficacy of vocabulary interventions over a similar intervention period have also found specific effects on researcher‐developed proximal measures rather than standard measures of vocabulary (Apthorp et al. 2012; Coyne et al. 2009, 2019; Elleman et al. 2009; Kim et al. 2021) and comprehension (e.g., Fien et al. 2011; Vadasy et al. 2015). It is possible that our intervention period was simply too short to generate larger transfer effects, or that such effects may have emerged if we had a later measurement timepoint. The standard assessments we selected may also not have been sensitive enough for measuring intervention efficacy for such a short intervention period (e.g., see discussion in Elleman et al. 2009). Standardized measures of vocabulary are designed to measure vocabulary growth over a wide developmental period rather than incremental change in general vocabulary knowledge over the short duration of the study. There is an increasing focus on the importance of replication within educational research (Kim 2019; Kraft 2020; Lortie‐Forgues and Inglis 2019), and our findings need to be replicated and extended. A future iteration of an audiobook intervention might also explore longer‐term outcomes or consider implementing a longer intervention period. Children also selected from a range of fiction and nonfiction books which may have provided different opportunities for vocabulary learning; future work may also aim to tease apart genre effects.

Another future direction is to isolate the effect of the scaffolded reading instruction. Because there was no condition with one‐on‐one scaffolding sessions without listening to audiobooks, it is unknown what role the text‐supplemented audiobooks specifically played in the positive effects on vocabulary in the Audiobooks+Scaffold group. Further work should explore whether vocabulary and scaffolding instruction paired with audiobooks, compared to with text‐based books or without books at all, is specifically effective for poor readers. Finally, future research should examine factors influencing intervention efficacy for lower SES students, particularly for the scaffolding component. Surprisingly, it had greater benefits for higher SES students, highlighting the need to adapt it for better support of lower SES learners.

## 5 Conclusion

Together, these results suggest that listening to text‐supplemented audiobooks, particularly when paired with scaffolded instructional support, may support vocabulary growth in both good and struggling readers. One‐on‐one scaffolding meetings also increased the amount of time children listened to the books during the study. The remote implementation of the intervention is promising from a scalability standpoint, as it was successfully administered during the Covid‐19 pandemic across the United States. Overall, this study suggests that audiobooks paired with instructional support are a promising potential solution for struggling readers, worthy of future research.

## Funding

This research was supported by the Chan Zuckerberg Initiative for the Reach Every Reader project (https://www.gse.harvard.edu/reach‐every‐reader), the National Science Foundation (Graduate Research Fellowship 1745302 to H.A.O.), and the National Institutes of Health (F32‐HD100064 to OO‐P; F32HD117580 to H.A.O.). *Learning Ally* supported the online advertising for recruiting participant families.

## Ethics Statement

This study has been approved by MIT's Committee on the Use of Humans as Experimental Subjects.

## Conflicts of Interest

The authors declare no conflicts of interest.

## Supporting information




**Supporting File 1**: desc70159‐sup‐0001‐SuppMat.pdf

## Data Availability

Data, analysis code, and key materials are publicly available on OSF at https://osf.io/zac9d/.
