# Supplementary material for: Remote Text‐Supplemented Audiobook Intervention Supports Children's Explicit and Incidental Vocabulary Learning
Source: Dev Sci. 2026 Mar 17;29(3):e70159. doi: 10.1111/desc.70159 (PMC12995855; doi:10.1111/desc.70159)
Supplement: Supplementary file 1 — Supporting File 1: desc70159‐sup‐0001‐SuppMat.pdf [file DESC-29-e70159-s001.pdf]

SUPPLEMENTARY MATERIALS

TABLE OF CONTENTS

INTERVENTION DETAILS *2*

Supplementary Table 1: Book tracks 2

Supplementary Table 2: Scaffold lesson plan summary 4

MEASUREMENT DETAILS *5*

Supplementary Figure 1: Proximal vocabulary assessments 5

Supplementary Table 3: Standardized and Proximal Measures 6

MODEL RESULTS *9*

Supplementary Table 4: Full Regression Models for Vocabulary Outcomes 9

Supplementary Table 5: Full Regression Models for Vocabulary Outcomes with Book Covariates 11

Supplementary Table 6: Main effect models without KBIT as a covariate 13

METHODS DETAILS *14*

Deviations from preregistration 14

Supplementary Table 7: Percents of missing data for variables included in the manuscript analyses 15

INTERVENTION DETAILS

Supplementary Table 1: Book tracks

Track 1	Track 2	Track 3
(all remaining)	Track Assignment:<br>3rd Grade: CELF<10<br>4th Grade: CELF<7 & PPVT<100	Track Assignment:<br>3rd Grade: CELF>=10<br><br>4th Grade: CELF<7 & PPVT>=100 OR CELF>=7 & PPVT<100 OR CELF>=7 & PPVT>=100
Primary:<br>1. *Hank Zipzer,* by Henry Winkler and Lin Oliver (**FIRST**) [750L]<br>2. *Memphis, Martin, and the Mountaintop : The Sanitation Strike of 1968*, by Alice Faye Duncan [800L]<br>3. *The Boy Who Invented TV : The Story of Philo Farnsworth*, by Kathleen Krull [860L]<br>4. *The Chocolate Touch*, by Patrick Skene Catling [770L]<br>5. *I Survived The Attack of the Grizzlies, 1967*, by Lauren Tarshis [550L]<br>6. *Who Was Maya Angelou?*, by Ellen Labrecque (**LAST**) [900L]<br><br>Additional titles:<br>● *How To Eat Fried Worms*, by Thomas Rockwell [560L]<br>● *The One And Only Ivan*, by Katherine Applegate [570L]<br>● *The Mystery of the Missing Cat*, by Gertrude Chandler Warner [530L]	Primary:<br>1. *Mr. Klutz Is Nuts*, by Dan Gutman (**FIRST**) [700L]<br>2. *Amina's Voice*, by Hena Khan [800L]<br>3. *Who Was Galileo?*, by Patricia Brennan Demuth [760L]<br>4. *Frindle*, by Andrew Clements [830L]<br>5. *Thirty Minutes Over Oregon: A Japanese Pilot's World War II Story*, by Marc Tyler Nobleman (**LAST**) [990L]<br><br>Additional titles:<br>● *The Lemonade War*, by Jacqueline Davies [630L]<br>● *We Are The Ship : The Story of Negro League Baseball*, by Kadir Nelson [900L]<br>● *Who Was Maya Angelou?*, by Ellen Labrecque [900L]<br>● *The Chocolate Touch*, by Patrick Skene Catling [770L]<br>● *Memphis, Martin, and the Mountaintop : The Sanitation Strike of 1968*, by Alice Faye Duncan [800L]<br>● *Tales Of A Fourth Grade Nothing*, by Judy Blume [470L]	Primary:<br>1. *Frindle*, by Andrew Clements (**FIRST**) [830L]<br>2. *Schomburg: The Man Who Built A Library*, by Carole Boston Weatherford [1100L]<br>3. *The Bad Beginning*, by Lemony Snicket [1010L]<br>4. *Puppies Dogs and Blue Northers: Reflections on Being Raised by a Pack of Sled Dogs*, by Gary Paulsen [1190L]<br>5. *Crenshaw*, by Katherine Applegate (**LAST**) [540L]<br><br>Additional titles:<br>● *Chasing Space Young Readers' Edition*, by Leland D. Melvin [1020L]<br>6. ● *Thirty Minutes Over Oregon: A Japanese Pilot's World War II Story*, by Marc Tyler Nobleman [990L]<br>● *We Are The Ship : The Story of Negro League Baseball*, by Kadir Nelson [900L]<br>● *Amina's Voice*, by Hena Khan [800L]<br>● *The Reptile Room*, by Lemony Snicket [1040L]<br>● *Bob*, by Wendy Mass [590L]<br>● *Young Captain Nemo,* by Jason Henderson [680L]

• *Tales Of A Fourth Grade Nothing*, by Judy Blume [470L]		• *Lifeboat 12*, by Susan Hood [610L]

Participants in the audiobook groups were assigned to a book track based on their grade, listening comprehension (CELF standard score), and receptive vocabulary (PPVT standard score). These assignments could change based on children's experiences with the first book. Typically, participants read the designated first book and last book; however, if children reported having read the book previously, they were tested on and read another book instead. These book tracks were created by the Learning Ally team for our study to include some grade level books and some above-grade-level books, balance fiction and nonfiction, include diverse authors and topics, and add up to a similar number of total minutes across the intervention period if all recommended books were completed. Lexile measures for each book are provided in brackets.

Supplementary Table 2: Scaffold lesson plan summary

Week	Session 1	Session 2
1	Sequencing	Comprehension Monitoring
2	Text Mapping Noun Phrases	Retelling
3	Main Character	Predicting
4	Text Mapping Verb Phrases	Author’s Purpose
5	Text Mapping Prefixes	Character’s Goals
6	Reporting	Prediction
7	Text Mapping - Comparing Characters	Sequencing
8	Alternate Outcomes	Alternate Outcomes

Lesson plans were adapted from the Language and Reading Research Consortium materials.

MEASUREMENT DETAILS

Supplementary Figure 1: Proximal vocabulary assessments

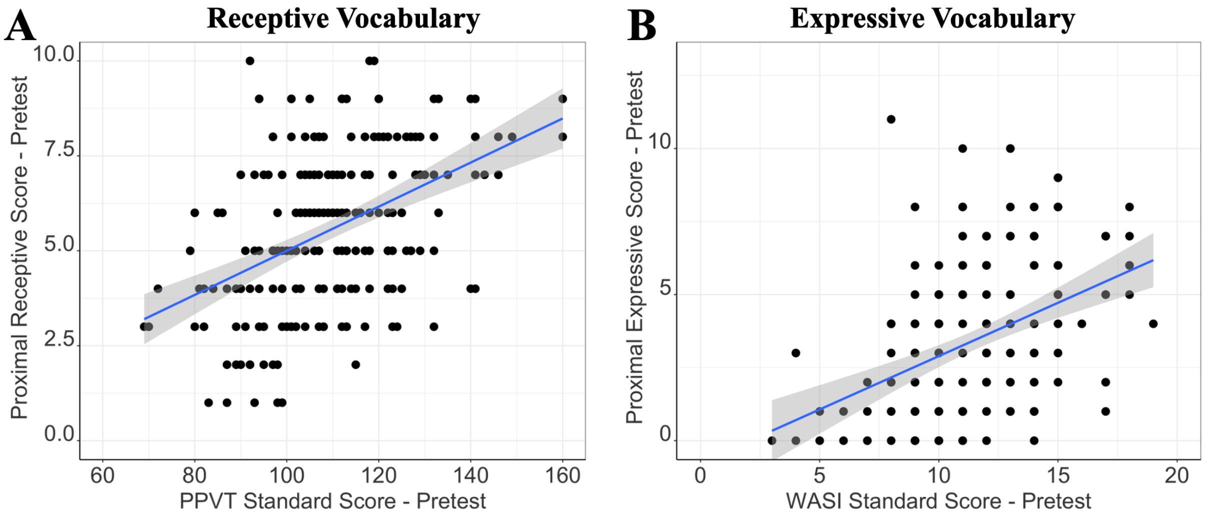

To determine whether our proximal vocabulary measures captured meaningful variation in children's vocabulary skills, we compared participants' pre-test scores for the two receptive vocabulary measures (PPVT raw score and final-book proximal receptive vocabulary score), and for the two expressive vocabulary measures (WASI vocabulary subtest raw score and final-l book proximal expressive vocabulary score). There was a positive correlation for the standard and proximal scores for both receptive (Pearson's correlation, r=.51, p<.001) and expressive (Pearson's correlation, r=.54, p<.001) vocabulary. Thus, we believe that the proximal measures are capturing some meaningful variation in children's vocabulary that is related to the standard measures they were based on. Scatterplots show raw scores for the standardized measures on the x-axis (PPVT; WASI) at pre-test, and scores for the proximal measures on the y-axis, for ***(A)*** *receptive vocabulary and* ***(B)*** *expressive vocabulary (using actual, not imputed data).*

Supplementary Table 3: Standardized and Proximal Measures

Measures	Description	Sample reliability coefficients
**Standardized measures**		
Clinical Evaluation of Language Fundamentals, 5th Edition (CELF-5) - Understanding Spoken Paragraphs	Standardized listening comprehension assessment. Participants listened to passages read aloud by the examiner and then answered a series of verbal questions related to the passages.	**Pretest** 0.807(0.775-0.835)
		**Posttest** 0.727(0.682-0.767)
Peabody Picture Vocabulary Test, 5th Edition (PPVT-5)	Standardized receptive vocabulary assessment. Participants were prompted with a word by the examiner and shown four pictures where they had to choose the one most closely related to the word.	**Pretest** 0.729(0.685-0.768)
		**Posttest** 0.852 0.828-0.874)
Wechsler Abbreviated Scale of Intelligence, Second Edition (WASI-II)	Standardized expressive vocabulary assessment. Participants were presented individual words by the examiner and asked to provide a verbal definition for each of the words.	**Pretest** 0.746(0.703-0.784)
		**Posttest** 0.676 (0.620-0.725)
Motivation to Read	Standardized survey for reading motivation. The examiner read a series of questions aloud to the participants, who then responded to evaluate their motivation to read.	**Pretest** 0.838 (0.811-862)
		**Posttest** 0.833 (0.805-858)
Kaufman Brief Intelligence Test, 2nd Edition (KBIT-2) - Matrices	Standardized non-vebal IQ assessment. Participants were shown a set of figures with a missing piece and asked to choose the option that would complete the pattern.	**Pretest** 0.83
Dynamic Indicators of Basic Literacy Skills (DIBELS) -	Standardized assessments used to assess reading skills.	Item level data was not available

1. Word Reading Fluency (WRF)<br>2. Passage Reading Fluency (PRF)<br>3. Multiple Choice Reading Comprehension (MCRC)	1. Participants were given one minute to read words from a list as quickly and as accurately as they could without making mistakes.<br>2. Participants were given one minute to read a passage as quickly and as accurately as they could without making mistakes.<br>3. Participants were instructed to read a passage and then answer corresponding multiple-choice questions. The examiner did not read the passages or questions aloud for this test.	
Comprehensive Test of Phonological Processing, 2nd Edition (CTOPP-2)<br>1. Non-word Repetition<br>2. Memory for digits<br>3. Blending words	Standardized assessments used to assess working memory skills.<br>1. Participants were presented with a non-word read by the examiner and asked to repeat it.<br>2. Participants were presented with a sequence of numbers and asked to repeat them in the same order.<br>3. Participants were presented with parts of a word and asked to repeat them back as a whole word	**Pretest**<br>0.7<br>0.8<br>0.84
**Proximal Measures**		
Proximal Expressive Vocabulary	Researcher-developed vocabulary assessment. This tool	**Pretest**<br>0.531(0.442-0.609)

	was developed as a book-specific assessment where participants were asked to define 10 words that appeared in the books they were assigned to read either at the beginning or end of the intervention *(modeled after the WASI-2)*	**Posttest** 0.504(0.408-0.588)
Proximal Receptive Vocabulary	Researcher-developed vocabulary assessment. This tool was developed as a book-specific assessment where participants were presented with a word followed by 4 pictures. They were asked to select the picture that most closely related to the word. 14 words were selected from books that were read at the beginning or end of the intervention *(modeled after PPVT-5)*	**Pretest** 0.511(0.420-0.590)<br>**Posttest** 0.536(0.450-0.611)

Reliabilities are Cronbach's alpha with 95% confidence intervals in parentheses. Some reliabilities were calculated and reported in Ozernov-Palchik, Olson, et al., 2022.

MODEL RESULTS

Supplementary Table 4: Full Regression Models for Vocabulary Outcomes

Model: posttest_score ~ pretest_score + age + gender + KBIT + lowEd + PR + group

Outcome Measure	Predictor	Estimate	Std. Error	t	p	95% CI Lower	95% CI Upper
Proximal Receptive Vocabulary	(Intercept)	0.764	2.186	0.35	.727	-3.55	5.08
	Pretest score	0.394	0.069	5.73	< .001	0.26	0.53
	Age	0.214	0.187	1.15	.254	-0.15	0.58
	Gender	-0.020	0.196	-0.10	.918	-0.41	0.37
	KBIT standard score	0.008	0.008	0.92	.358	-0.01	0.02
	Poor reader	-0.254	0.222	-1.15	.254	-0.69	0.18
	Low parental education	-0.444	0.228	-1.95	.054	-0.90	0.01
	Audio > Mind	0.238	0.250	0.95	.343	-0.26	0.73
	Scaffold > Mind	1.010	0.252	4.01	< .001	0.51	1.51
Proximal Expressive Vocabulary	(Intercept)	-3.108	2.757	-1.13	.262	-8.57	2.35
	Pretest score	0.527	0.068	7.72	< .001	0.39	0.66
	Age	0.410	0.259	1.58	.118	-0.11	0.93
	Gender	-0.321	0.242	-1.33	.187	-0.80	0.16
	KBIT standard score	0.008	0.009	0.86	.389	-0.01	0.03
	Poor reader	-0.347	0.286	-1.21	.228	-0.91	0.22
	Low parental education	-0.510	0.281	-1.81	.073	-1.07	0.05
	Audio > Mind	0.764	0.304	2.51	.013	0.16	1.37
	Scaffold > Mind	1.636	0.314	5.21	< .001	1.01	2.26
Standard Receptive Vocabulary (PPVT)	(Intercept)	26.940	19.187	1.40	.163	-11.00	64.88
	Pretest score	0.473	0.053	8.92	< .001	0.37	0.58
	Age	5.332	1.813	2.94	.004	1.72	8.94
	Gender	0.580	1.748	0.33	.741	-2.87	4.03
	KBIT standard score	0.152	0.067	2.26	.025	0.02	0.28
	Poor reader	-0.672	2.067	-0.32	.746	-4.77	3.43
	Low parental education	-3.176	1.927	-1.65	.102	-6.99	0.64
	Audio > Mind	-1.541	2.113	-0.73	.467	-5.72	2.64
	Scaffold > Mind	-0.888	2.078	-0.43	.670	-4.99	3.22
Standard Expressive Vocabulary (WASI)	(Intercept)	4.089	5.160	0.79	.429	-6.11	14.28
	Pretest score	0.525	0.051	10.21	< .001	0.42	0.63
	Age	1.093	0.479	2.28	.024	0.14	2.04
	Gender	0.026	0.494	0.05	.958	-0.95	1.00
	KBIT standard score	0.005	0.019	0.28	.777	-0.03	0.04
	Poor reader	-0.814	0.568	-1.43	.154	-1.94	0.31
	Low parental education	-0.787	0.537	-1.47	.145	-1.85	0.28

	Audio > Mind	-0.133	0.566	-0.23	.815	-1.25	0.98
	Scaffold > Mind	-0.353	0.553	-0.64	.523	-1.44	0.74

Supplementary Table 5: Full Regression Models for Vocabulary Outcomes with Book Covariates

Model: posttest_score ~ pretest_score + age + gender + IQ + lowEd + bookTrack + FinalBook_Test_WeeksApart + Same_FinalBook + PR + group

Outcome Measure	Predictor	Estimate	Std. Error	t	p	95% CI Lower	95% CI Upper
Proximal Receptive Vocabulary	(Intercept)	0.114	2.591	0.04	0.965	-5.01	5.24
	Pretest score	0.432	0.074	5.87	0.000	0.29	0.58
	Age	0.311	0.217	1.43	0.154	-0.12	0.74
	Gender	0.197	0.214	0.92	0.358	-0.23	0.62
	KBIT standard score	0.008	0.009	0.93	0.356	-0.01	0.02
	Poor reader	-0.196	0.245	-0.80	0.425	-0.68	0.29
	Low parental education	-0.480	0.249	-1.93	0.055	-0.97	0.01
	Book track	-0.921	0.565	-1.63	0.107	-2.04	0.20
	Same final book	0.306	0.490	0.62	0.534	-0.67	1.28
	FinalBook_Test_WeeksApart	0.004	0.053	0.08	0.936	-0.10	0.11
	Audio > Mind	-0.005	0.280	-0.02	0.985	-0.56	0.55
	Scaffold > Mind	0.899	0.270	3.33	0.001	0.37	1.43
Proximal Expressive Vocabulary	(Intercept)	-1.348	2.824	-0.48	0.634	-6.93	4.23
	Pretest score	0.630	0.066	9.58	0.000	0.50	0.76
	Age	0.170	0.243	0.70	0.486	-0.31	0.65
	Gender	-0.206	0.235	-0.88	0.383	-0.67	0.26
	KBIT standard score	0.008	0.009	0.89	0.374	-0.01	0.03
	Poor reader	-0.140	0.268	-0.52	0.602	-0.67	0.39
	Low parental education	-0.466	0.272	-1.71	0.089	-1.00	0.07
	Book track	-0.710	0.609	-1.17	0.247	-1.92	0.50
	Same final book	0.491	0.553	0.89	0.378	-0.61	1.59
	FinalBook_Test_WeeksApart	0.046	0.056	0.81	0.418	-0.07	0.16
	Audio > Mind	0.695	0.317	2.19	0.030	0.07	1.32
	Scaffold > Mind	1.469	0.305	4.82	0.000	0.87	2.07
Standard Receptive Vocabulary (PPVT)	(Intercept)	8.362	16.962	0.49	0.623	-25.09	41.82
	Pretest score	0.642	0.046	13.87	0.000	0.55	0.73
	Age	4.045	1.436	2.82	0.005	1.21	6.88
	Gender	2.627	1.499	1.75	0.081	-0.33	5.58
	KBIT standard score	0.160	0.061	2.63	0.009	0.04	0.28
	Poor reader	-0.817	1.631	-0.50	0.617	-4.03	2.40
	Low parental education	-1.572	1.666	-0.94	0.347	-4.86	1.71
	Book track	-10.426	3.254	-3.20	0.002	-16.84	-4.01
	Same final book	4.867	2.828	1.72	0.087	-0.71	10.44
	FinalBook_Test_WeeksApart	0.643	0.353	1.82	0.070	-0.05	1.34
	Audio > Mind	-1.967	1.887	-1.04	0.298	-5.69	1.75
	Scaffold > Mind	-2.284	1.810	-1.26	0.208	-5.85	1.28
Standard Expressive Vocabulary (WASI)	(Intercept)	1.702	4.808	0.35	0.724	-7.78	11.18
	Pretest score	0.686	0.047	14.61	0.000	0.59	0.78
	Age	0.862	0.414	2.08	0.039	0.04	1.68
	Gender	0.154	0.420	0.37	0.715	-0.68	0.98
	KBIT standard score	-0.003	0.017	-0.15	0.882	-0.04	0.03
	Poor reader	-0.565	0.485	-1.17	0.245	-1.52	0.39
	Low parental education	-0.007	0.483	-0.01	0.988	-0.96	0.94
	Book track	-0.319	0.958	-0.33	0.740	-2.21	1.57
	Same final book	-0.390	0.832	-0.47	0.640	-2.03	1.25
	FinalBook_Test_WeeksApart	0.181	0.103	1.76	0.080	-0.02	0.38

	Audio > Mind	-0.153	0.545	-0.28	0.780	-1.23	0.92
	Scaffold > Mind	-0.701	0.529	-1.33	0.186	-1.74	0.34

Note: Because the imputed dataset did not include book tracking measures, only participants with these values are included in these model results.

Supplementary Table 6: Main effect models without KBIT as a covariate

Model: posttest_score ~ pretest_score + age + gender + lowEd + PR + group

Measure	Contrast	Est	S.E.	t	p	d
Proximal Receptive Vocabulary	Audio > Mind	0.233	0.250	0.93	.351	0.15
	Scaffold > Mind	0.939	0.255	3.68	< .001	0.59
Proximal Expressive Vocabulary	Audio > Mind	0.764	0.304	2.51	.013	0.35
	Scaffold > Mind	1.629	0.311	5.24	< .001	0.74
Standard Receptive Vocabulary (PPVT)	Audio > Mind	-1.519	2.122	-0.72	.475	-0.10
	Scaffold > Mind	-1.014	2.095	-0.48	.629	-0.06
Standard Expressive Vocabulary (WASI)	Audio > Mind	-0.111	0.563	-0.20	.844	-0.02
	Scaffold > Mind	-0.342	0.547	-0.63	.531	-0.07

METHODS DETAILS

Deviations from preregistration

Compared to the original preregistration, we generally followed the planned analyses for Aims 1 and 2, which examined the audiobook intervention's impact on vocabulary and comprehension, as well as the added benefit of scaffolded support. However, instead of using linear mixed-effects (LME) models, we employed robust linear models, as we determined they provided a more suitable analytic framework for our outcome measures and study design. Specifically, the *outcome = pretest × condition* approach (a) accounts for pre-existing differences in participants' baseline scores and (b) allows condition contrasts to be integrated more parsimoniously across the two outcome measures. For transparency, we provide the originally preregistered LME-based analyses and results on OSF (osf.io/zac9d/) to confirm that our findings remain robust under different analytic choices.

We diverged from the original plan for Aim 3. We did not implement structural equation modeling (SEM) to examine moderating effects of demographics, home learning environment, and COVID-related factors. Instead, our analysis focused on the moderating effects of socioeconomic status (SES)—as preregistered—and reading competency, which was newly introduced in this analytical framework.

Supplementary Table 7: Percents of missing data for variables included in the manuscript analyses

Variable	Missing %
Age	0.32
Gender	0
Grade Level	0
MCRC Pre	10.83
MCRC Post	20.7
CELF Listening comprehension Pre	0.32
CELF Listening comprehension Post	17.83
Receptive Vocabulary Pre	7.64
Receptive Vocabulary Post	17.52
PPVT Receptive Pre	1.27
PPVT Receptive Post	17.83
DIBELS PRF Pre	1.59
DIBELS PRF Post	19.43
Proximal Receptive Target Pre	10.83
Proximal Receptive Target Post	25.48
Parent Avg Education (years)	8.28
KBIT Score	0.32
Home Literacy Books	18.79
Proximal Expression Target Pre	10.19
Proximal Expression Target Post	22.93
DIBELS Word Reading Fluency	2.55
Race	8.92
Engagement Minutes	12.21
